# Natural Language Processing markers in first episode psychosis and people at clinical high-risk

**DOI:** 10.1038/s41398-021-01722-y

**Published:** 2021-12-13

**Authors:** Sarah E. Morgan, Kelly Diederen, Petra E. Vértes, Samantha H. Y. Ip, Bo Wang, Bethany Thompson, Arsime Demjaha, Andrea De Micheli, Dominic Oliver, Maria Liakata, Paolo Fusar-Poli, Tom J. Spencer, Philip McGuire

**Affiliations:** 1 Department of Computer Science and Technology, University of Cambridge, Cambridge, CB3 0FD UK; 2 Department of Psychiatry, University of Cambridge, Cambridge, CB2 0SZ UK; 3 The Alan Turing Institute, London, NW1 2DB UK; 4 Department of Psychosis Studies, Institute of Psychiatry, Psychology and Neuroscience, King’s College London, London, SE5 8AF UK; 5 Department of Public Health and Primary Care, University of Cambridge, Cambridge, CB1 8RN UK; 6 Department of Psychiatry, University of Oxford, Oxford, OX3 7JX UK; 7 Early Psychosis: Interventions and Clinical-detection (EPIC) Lab, Department of Psychosis Studies, Institute of Psychiatry, Psychology and Neuroscience, King’s College London, London, SE5 8AF UK; 8 Department of Brain and Behavioral Sciences, University of Pavia, Pavia, Italy; 9 School of Electronic Engineering and Computer Science, Queen Mary University London, London, E1 4NS UK; 10 OASIS service, South London and Maudsley NHS Foundation Trust, London, UK

**Keywords:** Diagnostic markers, Schizophrenia

## Abstract

Recent work has suggested that disorganised speech might be a powerful predictor of later psychotic illness in clinical high risk subjects. To that end, several automated measures to quantify disorganisation of transcribed speech have been proposed. However, it remains unclear which measures are most strongly associated with psychosis, how different measures are related to each other and what the best strategies are to collect speech data from participants. Here, we assessed whether twelve automated Natural Language Processing markers could differentiate transcribed speech excerpts from subjects at clinical high risk for psychosis, first episode psychosis patients and healthy control subjects (total *N* = 54). In-line with previous work, several measures showed significant differences between groups, including semantic coherence, speech graph connectivity and a measure of whether speech was on-topic, the latter of which outperformed the related measure of tangentiality. Most NLP measures examined were only weakly related to each other, suggesting they provide complementary information. Finally, we compared the ability of transcribed speech generated using different tasks to differentiate the groups. Speech generated from picture descriptions of the Thematic Apperception Test and a story re-telling task outperformed free speech, suggesting that choice of speech generation method may be an important consideration. Overall, quantitative speech markers represent a promising direction for future clinical applications.

## Introduction

Psychotic disorders typically develop at the end of adolescence or in early adulthood, following a clinical high risk (CHR-P) phase. Previous work has identified a number of clinical, cognitive, neuroimaging and peripheral blood measures that are associated with transition to psychosis in CHR-P subjects [1–4]. However, there remains a clinical need to develop more accurate predictive tools, which are non-invasive and can be easily translated to the clinic. Such methods could open the gateway to preventative interventions, targeted at those who need them most [5].

A core feature of psychotic disorders is Formal Thought Disorder, which is manifest as disorganised or incoherent speech. Recently, several automated approaches have been proposed to quantify speech disorganisation in transcribed speech from patients with psychotic disorders [6–12]. Elvevåg et al. [8] first proposed to use Latent Semantic Analysis (LSA) [13] to quantify semantic coherence of transcribed speech data from psychosis patients. Briefly, LSA represents each word as a vector, such that words used in similar contexts (e.g. ‘desk’ and ‘table’) were represented by similar vectors. Elvevåg et al. then used LSA to calculate the semantic coherence between adjacent words, the tangentiality of an individual’s speech, i.e. how likely it was to diverge off-topic over time, and semantic similarity between speech excerpts from different participants. Later work extended these approaches [6, 9], for example, to use new, state-of-the-art word and sentence embedding methods to obtain vectors from words and sentences, instead of LSA [9]. Other authors have used different approaches to quantify disorganised speech, such as automated measures of referential cohesion [9, 14], based on evidence this may be altered in patients with schizophrenia [15, 16]. Finally, Mota et al. [11] proposed a graph theoretical approach in which speech was represented as a graph. Speech graph connectivity was significantly reduced in patients with schizophrenia compared to healthy control subjects [11].

These automated approaches allow disorganised speech to be quantified and studied at scale. This is an important improvement on previous qualitative approaches which were subjective and time-consuming, limiting sample sizes. There is also growing evidence that quantitative speech markers can not only distinguish cases with psychosis and healthy controls [12, 17] but may help to predict the later onset of psychosis in CHR-P subjects. Corcoran et al. [7] reported that in a CHR-P sample, decreased semantic coherence (LSA), greater variance in semantic coherence, and reduced usage of possessive pronouns predicted transition to psychosis with approximately 80% accuracy. Rezaii et al. [18] predicted conversion to psychosis with approximately 90% accuracy from low semantic density and speech content focusing on voices and sounds. Mota et al. [10] obtained ~80% accuracy for predicting a schizophrenia diagnosis 6 months in advance, based on a speech graph approach [11].

While alterations in speech are an important component of psychosis, it is still unclear which strategies for assessing speech are most useful. For example, some studies analyse speech produced in response to a stimulus, while others examine free speech recorded during a conversation. In addition, to date, most studies have used a relatively limited set of measures to quantify disorganised speech, and there is a need to identify which analytic measures can provide a comprehensive overview of speech abnormalities in CHR-P individuals. Here, we aimed to address these questions in order to provide methodological insights into how best to quantify formal thought disorder in psychosis.

To that end, we first investigated whether twelve Natural Language Processing (NLP) measures could distinguish transcribed speech excerpts from CHR-P subjects, first episode psychosis (FEP) patients and healthy control subjects, using speech excerpts generated by asking participants to describe pictures from the Thematic Apperception Test (TAT; [19]). These pictures typically induce relatively incoherent speech in patients, and have been previously used both to assess thought disorder, for example with the Thought and Language Index assessment tool [20], and to identify the neural substrate of thought disorder [21, 22]. We also assessed whether NLP measures could distinguish CHR-P subjects who did or did not transition to psychosis. We included a range of NLP measures because these measures are computationally cheap to calculate (requiring at most a few seconds per participant, on a single CPU) and ultimately a combination of measures is likely to be more informative than any single measure. Ten of the NLP measures were chosen because they were widely employed in the prior literature, had been previously suggested to show differences in psychosis, and could plausibly capture a range of dimensions of thought disorder [6–9, 11, 12]. We also employed two additional measures: one potentially related to the repetitiveness of speech, motivated by prior evidence that perseverance is a component of thought disorder [20], and another of whether a participant’s speech was ‘on-topic’, which is related to tangentiality [8] and similar to measures previously employed by [8, 23]. Our motivation for these additional measures was to quantify aspects of thought disorder not already captured by the original ten metrics. Second, we investigated whether these NLP measures were correlated with each other, to explore whether they contained overlapping or complementary information and therefore might be usefully combined in future to predict conversion. Finally, we assessed whether speech generated using two alternative approaches to the TAT would show similar differences between the three participant groups, to ascertain which strategy for eliciting speech provided most power to assess thought disorder. In particular, we used speech generated by asking participants to re-tell stories from the Discourse Comprehension Test (DCT; [24]) and free speech excerpts.

## Materials and methods

### Participants

Three groups of participants were recruited as described by Demjaha et al. [25]: 25 CHR-P participants, 16 FEP patients and 13 healthy control subjects. CHR-P participants were recruited from the Outreach and Support in South London (OASIS) service [26], and met ultra-high risk criteria assessed with the Comprehensive Assessment of At-Risk Mental States (CAARMS; [27, 28]). FEP patients were recruited from the South London and Maudsley NHS Foundation Trust. Healthy controls with no previous or current history of psychiatric illness and no family history of psychosis were recruited from the same geographical area. Groups were matched for age (one-way ANOVA, *P* = 0.38) and sex (*P* = 0.33); see Table 1.

**Table 1:** Sample characteristics for the three groups: healthy control subjects (CON), clinical high risk subjects (CHR-P) and first episode psychosis patients (FEP).

	CON	CHR-P	FEP	Group difference
Sample size	13	25	16	*N/A*
Age (years)	26.5 ± 5.2	25.1 ± 4.8	24.5 ± 3.7	*P* = *0.38*
Sex (M)	8 (61.5%)	15 (62.5%)	13 (81.3%)	*P* = *0.33*
No. on antipsychotic medication	0	4	6	*P* = *0.031*
Years in education	18.4 ± 4.2	13.0 ± 2.8	13.3 ± 1.9	*P* < *0.001*
WRAT IQ	115.6 ± 5.2	103.3 ± 11.8	99.8 ± 15.0	*P* = *0.0019*
Digit span	20.7 ± 4.1	17.0 ± 3.6	13.3 ± 4.5	*P* < *0.001*
TLI total	0.37 ± 0.51	1.8 ± 1.4	3.5 ± 2.9	*P* < *0.001*
TLI positive	0.37 ± 0.51	1.4 ± 1.3	2.9 ± 3.0	*P* = *0.0029*
TLI negative	0 ± 0	0.27 ± 0.61	0.58 ± 0.86	*P* = *0.055*

We note that age information was missing for two participants: one CHR-P subject and one FEP patient and sex information was missing for one CHR-P subject. Results are reported as the mean average and standard deviation where appropriate. Group differences were calculated using a 1-way ANOVA. WRAT IQ, digit span, TLI and education information were missing for one CHR-P subject.

*TLI* Thought and Language Index, *WRAT IQ* Wide Range Achievement Test Intelligence Quotient.

All participants were fluent in English and gave written informed consent after receiving a complete description of the study. Ethical approval for the study was obtained from the Institute of Psychiatry Research Ethics Committee.

CHR-P subjects were followed clinically for an average of 7 years after participating in the study to assess whether they subsequently developed a psychotic disorder. Eight of the 25 CHR-P subjects transitioned to psychosis. Transition to psychosis was defined as the onset of frank psychotic symptoms that did not resolve within a week.

### Procedure

Our primary analyses were performed using transcribed speech generated using the Thematic Apperception Test (TAT; [19]). Participants were presented with eight TAT pictures and asked to talk about each picture for one minute. Pictures were presented in the same order to all participants. If the participant stopped talking during the minute they were prompted to continue, using the prompts: “Anything else?”, “What do you think is happening?”, “Can you describe it more fully?”. Speech samples were recorded and transcribed by a trained assessor blind to group status. Inaudible parts of speech were noted as [?] [29].

We repeated our analyses using speech data generated from the same participants with two alternative approaches. First, participants were read six stories from the Discourse Comprehension Test (DCT; [24]) and asked to re-tell them. Finally, free speech was recorded from an interview in which participants were asked to speak for 10 minutes about any subject. Participants often chose subjects such as their hobbies and interests, life events and plans for the weekend. If the participant stopped talking, they were prompted to continue, using a list of topics the participant was happy to discuss.

Data was not available for all participants for all tasks. For the TAT task, no data was available for 1 participant and 1 participant’s recording was excluded due to poor audio quality, leaving *N* = 52. A further 1 participant had 1 picture response (out of 8) missing and was included with only 7 picture descriptions. For the DCT task, 3 participants had no data available, leaving *N* = 51. 6 participants had 1 story response (out of 6) missing and 1 participant had 2 story responses missing; these participants were included with the responses available. For free speech, 2 participants had no data available, leaving *N* = 52. Tasks were presented in the same order to all participants, with the free speech task first, then the TAT task and the DCT task.

Thought disorder was assessed by applying the Thought and Language Index (TLI; [20]) to the TAT speech excerpts, again by a trained assessor blind to group status. The positive and negative syndrome scale (PANSS; [30]) was used to measure symptoms. Participants also completed the WRAT IQ test [31], the Wechsler Adult Intelligence Scale Digit Span test [32], and reported the number of years they spent in education.

### Natural Language Processing measures

#### Basic measures

For each excerpt, we calculated the total number of words, *N*_word_, the total number of sentences, *N*_sent_, and the mean number of words per sentence, *N*_word_/*N*_sent_.

#### Semantic coherence

Speech incoherence was conceptualised by [33] as “a pattern of speech that is essentially incomprehensible at times”, and [34] later linked to problems integrating meaning across clauses [35]. Here we quantified semantic coherence using the same approach as [6, 9], which measures how coherent transcribed speech is in terms of the conceptual overlap between adjacent sentences. The text was first split into sentences and pre-processed by removing stop words (defined from the NLTK corpus [36]) and filler words (e.g. ‘um’). Each remaining word was then represented as a vector, using word embeddings from the word2vec pre-trained Google News model [37]. From these word embeddings, we calculated a single vector for each sentence, using Smooth Inverse Frequency (SIF) sentence embedding [38]. We used word2vec and SIF embeddings because they previously gave the greatest group differences between patients with schizophrenia and control subjects [9]. Finally, having represented each sentence as a vector, the semantic coherence was given by the mean cosine similarity between adjacent sentences [6, 9].

#### Tangentiality

Tangentiality captures the tendency of a subject to drift ‘off-topic’ during discourse. We used the tangentiality measure described by [8, 9], where, for a given response, the cosine similarity was calculated between each sentence in the participant’s response and an a priori description of the stimulus used to generate speech (e.g. a sentence describing the TAT picture). Again, we used word2vec and SIF for word and sentence embeddings, respectively. Tangentiality was then computed as the slope of the linear regression of the cosine similarities over time (ranging from −1 to 1). A more negative slope means the response became less closely related to the stimulus over time.

For the TAT task, we used a priori descriptions of each of the 8 pictures from [39]; see Section S1. For the DCT task we used the original stories to calculate the a priori vectors. Note that we did not obtain tangentiality scores from free speech, due to the absence of an a priori description.

#### On-topic score

We also employed an ‘on-topic’ score, which is closely related to tangentiality. Here, instead of calculating the slope of the cosine similarities over time, we calculated the mean of the cosine similarities between each sentence and the a priori stimulus description (ranging from −1 to 1). This measure captures how ‘on-topic’ the participant’s response to the stimulus was on average across the whole response, rather than whether it became less closely related to the stimulus over time. The measure is similar to the approach used by [23] where LSA vectors representing participants’ descriptions of a story were compared with a vector representing the original story. Again, we used the TAT picture descriptions from [39] and the original DCT stories as the a priori descriptions, and we did not obtain on-topic scores for free speech.

#### Repetition

Prior work has suggested that speech from patients with schizophrenia may be more repetitive than control subjects [20]. As a first step towards measuring repetitiveness quantitatively, we calculated the cosine similarity between all possible pairs of sentences, and defined a candidate repetition score as the maximum cosine similarity between any two sentences (ranging from −1 to 1). A maximum similarity score of 1 means that (at least) two of the sentences in the response were represented by identical vectors, suggesting the same content was repeated.

#### Number of ambiguous pronouns

Given evidence that patients with schizophrenia may not use referential pronouns correctly [16, 9] proposed to count the number of ambiguous pronouns as a syntactic measure of speech incoherence. Here, ambiguous pronouns are pronouns which were either (1) never resolved (e.g. “I think that’s *their* dog”, where “they” are never named) or (2) resolved only after the use of a proper noun (e.g. “I told *him* to go away, *my friend*, I didn’t want to see him”) [9]. Following [9], we first identified all the pronouns in a participant’s response and the subject they referred to, using a pre-trained co-reference resolution model [40]. We then counted the number of times the first term used to refer to a subject was a third-person pronoun (he, she, etc).

#### Speech graphs

Speech graphs were proposed by [12]. Briefly, each unique word in a participant’s response is represented by a node, and directed edges link the words in the order in which they were spoken. Prior work has already applied speech graph analysis to our TAT speech excerpts [29], and found significant group differences in speech graph connectivity. Here, we compared speech graph connectivity to the other NLP measures above. We also applied the speech graph approach to speech from the DCT task, and free speech.

Following [29], we used the SpeechGraphs software [11] to calculate four measures of graph connectivity: the total number of nodes in the largest connected component (LCC) and the largest strongly connected component (LSC) [10, 11], plus the corresponding values normalised to randomised speech graphs- LCCr and LSCr [11, 29]; see Section S3.

### Statistical analyses

The metrics described above were calculated for each speech excerpt. Where there was more than one excerpt available per subject (e.g. from 8 TAT pictures), we calculated the mean score across the excerpts, to obtain a single value per subject.

We used the Shapiro-Wilk test to assess the Normality of the NLP measures, see Table S1. Some measures were not Normally distributed, and we used the two-sided Mann–Whitney *U*-test to calculate the statistical significance of group differences. The relationships between different NLP measures were calculated with linear regression, controlling for group membership as a co-variate.

We counted the number of inaudible pieces of speech in each excerpt, normalised to the total number of words. We assessed whether there were significant differences in the number of inaudible pieces of speech per word between groups or between the TAT, DCT and free speech methods using the two-sided Mann–Whitney *U*-test. For those methods where there were differences, as an additional sensitivity analysis we tested whether group differences in the NLP metrics remained significant when controlling for the number of inaudible pieces of speech per word, using a Generalized Additive Model for Location, Scale and Shape (GAMLSS) with a gamma distribution [41].

We also used GAMLSS models to control for IQ, years in education and digit span test score. For these post-hoc sensitivity analyses, we report multiplicative effect sizes on the mean (*λ*) in addition to *T*-statistics and *P*-values.

## Results

### Speech profiles

**Fig. 1:**
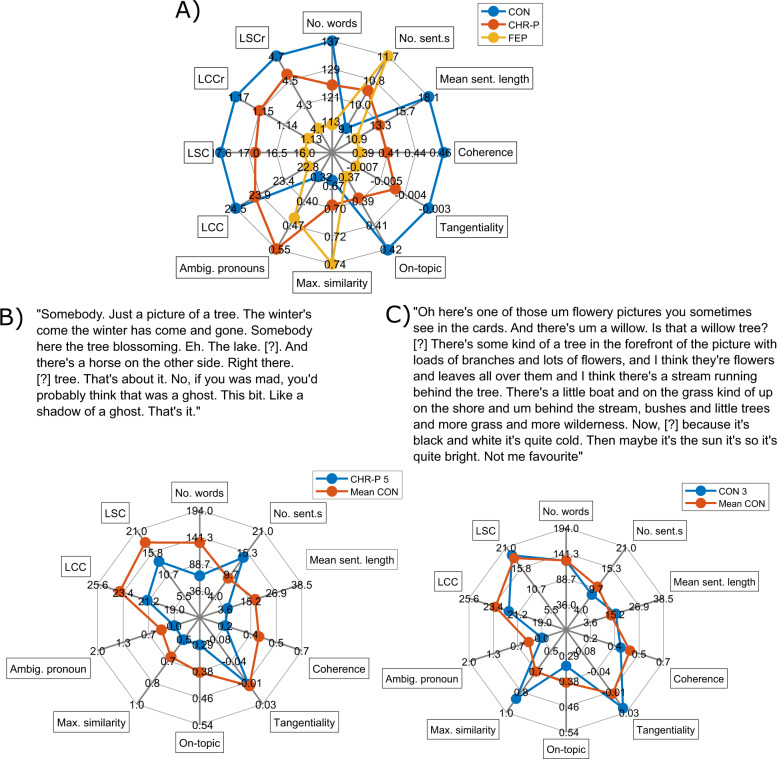
Speech profiles. **A** Average speech profiles for the control subjects, CHR-P subjects and FEP patients. **B**, **C** Example descriptions of one of the TAT pictures, for a particular CHR-P subject and control subject, respectively. The response in part **B** diverges somewhat from the average control response, with more, shorter sentences, and lower coherence, on-topic score and LCC, for example. The response in part **C** follows the average control response quite closely, but has a somewhat higher maximum similarity between sentences. We note that the healthy control subject whose speech profile is given in part **C** was excluded from our calculation of the average control response, to avoid inflating the similarity between their speech profile and the average control profile. Spider plots were generated using code from ref. [48].

We first calculated all twelve NLP measures outlined in the ‘Methods’ section, for the TAT excerpts from all subjects. The average values for all measures per group are shown as average ‘speech profiles’ (spider plots) in Fig. 1A. For illustrative purposes, in Fig. 1B, C we show speech profiles for two participants’ descriptions of one of the TAT pictures.

### Group differences in NLP measures, for the TAT

**Fig. 2:**
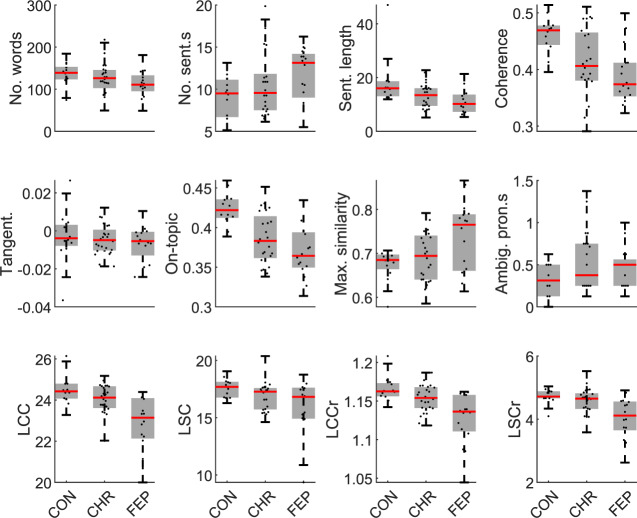
Box-plots showing group differences in all twelve NLP measures. Results are shown for speech generated using the TAT.

Table 2 gives group differences for all NLP measures obtained from the TAT speech excerpts, with corresponding box-plots in Fig. 2. Comparing FEP patients to control subjects, both number of words and mean sentence length were significantly lower for FEP patients, whilst the number of sentences was significantly higher. We also observed lower semantic coherence for FEP patients, in-line with [9]. Tangentiality did not show any significant group differences, however on-topic score significantly decreased in FEP patients, showing a larger group difference than any other measure. This suggests that FEP patients’ responses did not diverge from the prior picture description over time, but were instead less closely related to the prior picture description on average across all time points. There were no significant differences in the ambiguous pronoun count between the FEP patients and control subjects, in contrast to [9], or in the maximum similarity (repetition) measure. As previously reported [29], speech graph connectivity was reduced in FEP patients, in-line with [10, 11].

**Table 2:** Statistical group differences in NLP measures.

	TAT			DCT			Free		
	FEP/CON	CHR-P/CON	FEP/CHR-P	FEP/CON	CHR-P/CON	FEP/CHR-P	FEP/CON	CHR-P/CON	FEP/CHR-P
*N*_word_	**−2.2 (0.027)**	−1.1 (0.26)	−1.3 (0.19)	**−2.3 (0.020)**	−1.2 (0.25)	−1.4 (0.17)	0.50 (0.62)	−0.19 (0.85)	0.48 (0.63)
*N*_sentence_	**2.2 (0.031)**	0.77 (0.44)	1.1 (0.26)	0.61 (0.54)	−0.65 (0.52)	1.2 (0.25)	**2.6 (0.0093)**	0.56 (0.57)	**2.3 (0.024)**
Sentence length	**−3.0 (0.0028)**	−1.9 (0.054)	−1.7 (0.095)	**−2.8 (0.0056)**	0 (1)	**−2.8 (0.0056)**	−0.98 (0.33)	−0.14 (0.89)	−1.8 (0.073)
Coherence	**−3.3 (<0.001)**	**−2.3 (0.022)**	−1.3 (0.19)	**−3.0 (0.0024)**	−1.1 (0.28)	**−2.4 (0.017)**	−1.8 (0.070)	−1.3 (0.20)	−0.87 (0.39)
Tangentiality	−0.95 (0.34)	−0.69 (0.49)	−0.23 (0.81)	0.76 (0.45)	−0.21 (0.83)	1.2 (0.22)	N/A	N/A	N/A
On-topic	**−3.5 (<0.001)**	**−3.1 (0.0017)**	−1.3 (0.20)	**−3.2 (0.0013)**	−1.6 (0.10)	**−2.0 (0.049)**	N/A	N/A	N/A
Maximum similarity	1.7 (0.082)	0.65 (0.51)	1.7 (0.090)	−0.72 (0.47)	0.50 (0.61)	−1.3 (0.20)	1.6 (0.10)	0.60 (0.55)	1.3 (0.20)
Ambig. Pronouns	1.2 (0.25)	1.8 (0.073)	−0.66 (0.51)	**2.3 (0.021)**	1.5 (0.14)	1.1 (0.28)	−0.75 (0.45)	−1.2 (0.23)	0.20 (0.84)
LCC	**−3.2 (0.0013)**	−1.5 (0.14)	**−2.9 (0.0033)**	**−3.0 (0.0028)**	−1.3 (0.18)	**−2.5 (0.014)**	−1.0 (0.31)	0.77 (0.44)	**−2.1 (0.037)**
LSC	−1.8 (0.067)	−1.7 (0.090)	−0.95 (0.34)	**−2.9 (0.0037)**	−1.1 (0.26)	**−2.4 (0.018)**	0.41 (0.68)	1.3 (0.19)	−0.90 (0.37)
LCCr	**−3.4 (<0.001)**	−1.7 (0.084)	**−2.6 (0.0091)**	**−2.6 (0.0011)**	−1.3 (0.20)	−1.9 (0.057)	−1.0 (0.31)	0.55 (0.58)	**−2.0 (0.049)**
LSCr	**−3.3 (<0.001)**	−1.4 (0.17)	**−2.6 (0.0091)**	**−3.3 (<0.001)**	**−2.0 (0.046)**	**−2.7 (0.0075)**	−0.72 (0.47)	1.2 (0.23)	**−2.0 (0.042)**

Z-values are given from Mann–Whitney *U*-tests, with the corresponding *P*-values in brackets. Results where *P* < 0.05 are highlighted in bold. LCC, LSC, LCCr, and LSCr results for the TAT have already been reported by ref. [29].

In the CHR-P group, on-topic score and semantic coherence were reduced compared to the control subjects. These measures showed no significant differences between CHR-P subjects and FEP patients. In contrast, LCC, LCCr and LSCr increased in CHR-P subjects with respect to FEP patients, but showed no significant differences between CHR-P subjects and control subjects.

4 of the CHR-P subjects and 6 of the FEP patients were taking antipsychotic medication (Table 1). Excluding subjects who were taking antipsychotic medication did not qualitatively change the group differences in the NLP measures; see Table S2, apart from the group difference in number of words between controls and FEP patients, which was no longer significant (*Z* = −1.7, *P* = 0.081).

When controlling for IQ, there were significant differences in LSC and LSCr between the CHR-P subjects who did or did not transition to psychosis (*T* = −2.8, *P* = 0.011 and *T* = −3.1, *P* = 0.0050, respectively). None of the other NLP measures differed between these two subgroups; see Table S3. These differences were not evident when not controlling for IQ.

### Number of prompts

Table S4 reports group differences in the number of prompts given to participants when describing the TAT pictures. FEP patients were given more prompts than both healthy control subjects (*Z* = 2.6, *P* = 0.0084) and CHR-P subjects (*Z* = 2.3, *P* = 0.020).

### Inaudible pieces of speech

For the TAT speech excerpts, there were no significant differences in the number of inaudible pieces of speech per word between the FEP patients and the control subjects (*Z* = 1.1, *P* = 0.26), or between the FEP patients and the CHR-P subjects (*Z* = −1.2, *P* = 0.22); Fig. S1. However, there was a significant difference in the number of inaudible pieces of speech per word between the CHR-P subjects and the healthy control subjects (*Z* = 2.2, *P* = 0.029); Table S5. All previously identified group differences in NLP metrics remained significant when controlling for the number of inaudible pieces of speech per word; see Table S6.

### Relationships between NLP measures

**Fig. 3:**
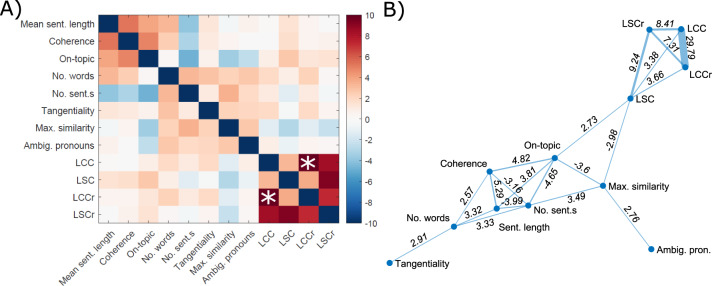
Relationships between NLP measures. **A** Heat mapping showing the relationships (T-statistics) between different NLP measures, calculated using linear regression, controlling for group membership. Colormap from ref. [49]. **B** Network showing the NLP measures which are significantly associated with each other, with *P* < 0.01. Corresponding T-statistics are shown on the network edges between measures. *The colorbar was truncated at *T* = 10 for visualisation purposes; *T* = 29.79 for the relationship between LCC and LCCr.

We next explored whether the NLP measures were significantly associated with each other, by fitting a linear regression model to each pair of NLP measures, controlling for group as a co-variate. Fig. 3A) shows the relationships between the NLP measures, with those that were significant with *P* < 0.01 plotted in the network in Fig. 3B).

The four speech graph measures (LCC, LCCr, LSC and LSCr) were strongly associated with each other, as expected. There was also a significant negative association between LSC and maximum similarity (repetition), and a significant positive association between LSC and on-topic score. Interestingly, there was no significant association between any of the speech graph measures and semantic coherence. Semantic coherence was significantly negatively associated with number of sentences and significantly positively associated with number of words, sentence length and on-topic score.

### Relationships between NLP measures and the TLI, symptoms and cognitive measures

We observed group differences in the TLI, IQ, number of years in education and the digit span test score; see Table 1. 15 CHR-P subjects and 8 FEP patients also had PANSS data available. Table S7 shows the associations between the NLP measures and the TLI, PANSS, IQ and number of years in education. After FDR correction for multiple comparisons (12 × 8 = 96 comparisons across all NLP and TLI, symptom and cognitive measures), we observed significant associations between TLI negative and: the number of words (*T* = −4.9, *P*_*FDR*_ < 0.001), LCC (*T* = −4.1, *P*_*FDR*_ = 0.0038), LCCr (*T* = −5.4, *P*_*FDR*_ < 0.001), LSC (*T* = −4.4, *P*_*FDR*_ = 0.0023) and LSCr (*T* = −3.6, *P*_*FDR*_ = 0.014).

There were no significant associations between NLP measures and IQ or number of years in education (although we note the potential for type 2 error given the small sample size and multiple comparisons correction- see Limitations). Nonetheless, after controlling for IQ and number of years in education as covariates in the GAMLSS models, not all group differences remained significant; see Tables S8 and S9 for T-statistics, *P*-values and effect sizes. In particular, between FEP patients and controls, the group differences in number of words, number of sentences and LSCr were no longer significant, although mean sentence length and other speech graph, coherence and on-topic measures still showed significant differences. The NLP metric whose multiplicative effect differed most from 1 was mean sentence length (*λ* = 0.77 controlling for years in education, *λ* = 0.82 controlling for IQ). Between CHR-P subjects and controls, group differences in coherence and, when controlling for education, on-topic score were no longer significant.

For the TAT task, there was a significant association between digit span test score and semantic coherence (Table S10; FDR corrected for 12 multiple comparisons as part of a post-hoc test). When controlling for digit span test score, only group differences in on-topic score and speech graph connectivity measures remained significant (see Table S11 for T-statistics, *P*-values and effect sizes).

### DCT task and free speech

Finally, we re-calculated the group differences for each of the NLP measures using speech generated from either the DCT story retelling task or free speech. Results are shown in Table 2. With the DCT task, we observed a significant decrease in semantic coherence and on-topic score in FEP patients with respect to healthy controls, as well as in the number of words, mean sentence length, LCC, LCCr, and LSCr, replicating the equivalent results for the TAT task. All of these measures apart from number of words and LCCr also showed significant reductions in FEP patients with respect to CHR-P subjects, but there were no significant differences between CHR-P subjects and healthy control subjects apart from for LSCr (unlike the TAT task where semantic coherence and on-topic score showed significant differences between CHR-P and control subjects, but not between CHR-P and FEP patients). With the DCT task we also observed a significant increase in the number of ambiguous pronouns in FEP patients with respect to control subjects, but there was no difference in ambiguous pronoun count between CHR-P subjects and either FEP patients or healthy controls.

With free speech, we observed a significant increase in the number of sentences spoken by FEP patients with respect to both CHR-P subjects and healthy controls. However, none of the other measures showed significant differences between FEP patients and healthy control subjects, including semantic coherence, on-topic score and maximum similarity. We note that the maximum similarity measure gave the highest possible score of 1 for several of the free speech excerpts, unlike for the TAT and DCT. This was due to the greater length of the free speech excerpts compared to the TAT and DCT excerpts, and suggests the measure may need adapting for use with longer excerpts. Interestingly, we did observe a significant decrease in LCC, LCCr, and LSCr in FEP patients with respect to CHR-P subjects, despite there being no significant difference between these measures for FEP patients and healthy controls.

For the DCT task, we observed significant correlations between the digit span test score and number of sentences, on-topic score and ambiguous pronoun count (Table S12). When controlling for digit span test score, no NLP group differences were statistically significant; see Table S13 for T-statistics, P-values and effect sizes.

There were no group differences in number of inaudible pieces of speech per word for the free speech excerpts, although there was a significant increase in number of inaudible pieces of speech per word for the FEP patients compared to control subjects for the DCT speech excerpts (*Z* = 2.0, *P* = 0.047). All previously identified group differences in NLP metrics observed from the DCT excerpts remained significant when controlling for the number of inaudible pieces of speech per word with the GAMLSS model, apart from the decrease in total number of words observed in the FEP patients compared to the healthy controls which was no longer significant (*Z* = −0.28, *P* = 0.78), and the difference in ambiguous pronoun count between FEP patients and healthy controls, which we were not able to test with the GAMLSS model; see Table S14. Whilst there was no significant difference in number of inaudible pieces of speech per word between the TAT and DCT speech excerpts, we did observe a significant reduction in number of inaudible pieces of speech per word in the free speech excerpts compared to both the TAT (*Z* = −3.1, *P* = 0.0022) and the DCT excerpts (*Z* = −4.0, *P* < 0.001), see Table S15; Fig. S2.

## Discussion

Our primary analysis of the TAT picture speech excerpts showed that several NLP measures did indeed discriminate between groups. Notably, both semantic coherence [9] and speech graph connectivity [11, 12] were significantly reduced in FEP patients compared to control subjects. Semantic coherence and speech graph connectivity also distinguished CHR-P subjects from control subjects and FEP patients, respectively (although the former was not robust to controlling for years in education), and speech graph connectivity was the only measure to show differences between CHR-P subjects who did or did not transition to psychosis (although only when controlling for IQ). There were no significant group differences in our novel measure of repetition or ambiguous pronoun count, although the latter may be worth re-visiting with more accurate co-reference resolution models as they become available. Interestingly, on-topic score exhibited significant group differences between control subjects and both CHR-P subjects and FEP patients, in contrast to the related measure of tangentiality [8, 9].

Given the small sample size, group differences in semantic coherence, sentence length and on-topic score between FEP patients and controls were remarkably robust to controlling for the potentially confounding effects of IQ and years in education. However, after controlling for IQ or years in education, the group difference in LSCr between FEP patients and controls was reduced, in-line with prior work showing that LSC varies with both IQ in normal development [42] and with educational level [43].

Second, we investigated the relationships between different NLP measures. There were some significant relationships, for example, we observed a negative association between LSC speech graph connectivity and the maximum similarity measure, which makes sense given that repetitive speech with fewer unique words will lead to fewer nodes being included in a speech graph and hence reduced connectivity. The ‘on-topic’ measure was positively related to semantic coherence and the LSC speech graph connectivity. Nonetheless, most inter-measure relationships were weak, for example there was no significant association between speech graph connectivity and semantic coherence.

These results suggest that different NLP measures may provide complementary information. It is predictable that different speech measures may capture distinct aspects of psychosis, e.g. different symptoms. Combining different measures in machine learning algorithms might also give additional power to predict future disease trajectories for CHR-P subjects, compared to using a single measure. Future studies should examine multiple NLP measures concurrently in larger samples, to test these hypotheses. The limited associations between the NLP measures and the TLI is also interesting and merits further consideration. The low computational cost of calculating the automated NLP measures described in this paper (at most seconds per participant) makes extracting multiple measures computationally straightforward.

Finally, we explored the impact of using different approaches to generate speech. Speech generated using the DCT story task replicated many of the NLP group differences observed with the TAT pictures. Free speech exhibited fewer, weaker NLP group differences compared to speech generated using the TAT pictures or the DCT story task, suggesting that this approach may be less sensitive for assessing thought disorder. A task-dependency is in-line with previous work, which found speech in which participants described their dreams was more predictive of psychosis than speech in which participants described their waking activities [11]. We note that the three tasks had different cognitive demands (for example regarding working memory and executive function), which could be related to the differences in NLP metrics observed. We were unable to generate all NLP measures from free speech excerpts, for example due to a lack of a priori stimulus description from which to calculate on-topic scores. These observations suggest that the task(s) used to generate speech in future studies should be considered carefully.

### Limitations

Ultimately, further external work is required before speech measures are ready to be “rolled out” to clinical applications.

A key limitation of this study was the sample size, which was in-line with prior work, but still small considering the known heterogeneity of CHR-P subjects [44]. The number of CHR-P subjects who transitioned to psychosis (*N* = 8) was therefore correspondingly small. The modest sample size means that there is a potential risk of type 2 errors. Further work is also needed to test the generalisability of our findings, and replicate them in larger cohorts of CHR-P subjects. To facilitate such work, we have made our code openly available on GitHub: https://github.com/SarahMorgan/NLP_psychosis.

The modest sample size meant we focussed on group-level, statistical analyses. However, to be clinically useful, future work will need to use NLP measures to predict individual disease outcomes, for example by applying more “data hungry” machine learning approaches. We believe our results provide an important step towards large studies at the individual level, by highlighting which methods may be best suited to eliciting incoherent speech and the potential power of combining multiple NLP measures.

The present study focused on FEP patients, and did not include patients with chronic psychosis. Consequently, we were not able to examine how acute FTD may differ from chronic FTD [45, 46]. This would be important to address in future work using automated NLP markers of transcribed speech. We focussed on 12 NLP measures but there are many more that may show significant group differences, e.g. pronoun incidence [47].

Finally, group comparison studies are vulnerable to differences in confounding factors between groups and here there were group differences in antipsychotic medication, IQ, number of years of education, working memory as assessed by the digit span test and number of prompts given (Tables 1 and S11). Excluding subjects who had been prescribed antipsychotic medication did not qualitatively change our main results (Section S5). Not all NLP group differences remained significant when controlling for IQ, years in education or digit span test score (Tables S3, S4, S12–15, effect sizes also provided). Most notably, when controlling for digit span for the DCT task, no NLP group differences were significant. In contrast, for the TAT task, group differences in on-topic score and speech graph connectivity remained significant after controlling for digit span, suggesting that the specific cognitive demands of the task are important. These task differences could suggest potential mechanisms. Future work should assess these relationships and task differences in more depth and investigate whether automated language markers provide additional predictive power beyond measures of cognition. It seems likely that group differences in the number of prompts reflected differences in the subjects’ speech rather than differences in how often they were prompted by the investigator, given that subjects were only prompted if they stopped speaking. Nonetheless, we cannot completely rule out the possibility that these or other, unobserved confounding factors might contribute to differences in NLP measures between groups. There were also significantly more inaudible pieces of speech per word in the free speech excerpts compared to the TAT and DCT excerpts, and the order in which tasks were presented to subjects was not randomized, which may be related to the weaker group differences in NLP metrics observed in the free speech excerpts.

## Conclusions

Overall, automated approaches to assessing disorganised speech show substantial promise for diagnostic applications. Quantifying incoherent speech may also give fresh insights into how this core symptom of psychotic disorders manifests.

## Supplementary information

Supplementary Information
