# Supplementary material for: Natural Language Processing markers in first episode psychosis and people at clinical high-risk
Source: Transl Psychiatry. 2021 Dec 13;11:630. doi: 10.1038/s41398-021-01722-y (PMC8669009; doi:10.1038/s41398-021-01722-y)
Supplement: Supplementary file 1 — Supplementary Information [file 41398_2021_1722_MOESM1_ESM.pdf]

Natural Language Processing markers in first episode psychosis and people at clinical high-risk - Supplementary Information

Sarah E Morgan, Kelly Diederen, Petra E Vértes, Samantha Ip, Bo Wang, Bethany Thompson, Arsime Demjaha, Andrea De Micheli, Dominic Oliver, Maria Liakata, Paolo Fusar-Poli, Tom J Spencer*, Philip McGuire*

October 14, 2021

Contents

1 TAT Picture Descriptions 2

2 Data collection details 2
- 2.1 DCT story task . . . 2
- 2.2 Free speech task . . . 2

3 Speech graph measure details 2

4 Normality 3

5 Antipsychotic medication 3

6 Transition to psychosis 4

7 Number of prompts 4

8 Inaudible pieces of speech 4

9 Relationships between NLP measures and the TLI, PANSS scores and cognitive measures 6

10 Education and IQ 8

11 Digit span test scores 8

12 DCT results 10
- 12.1 Digit span test scores . . . 10
- 12.2 Inaudible pieces of speech . . . 10

*TJS and PM share joint last authorship

1 TAT Picture Descriptions

TAT picture descriptions were taken from [1], and are copied below for reference.

Picture 6GF: An older man with a pipe in his mouth is talking to a younger woman sitting on a couch, who is looking back at him.

Picture 7GF: A young girl is sitting on a couch with a doll in her hands, and an older woman sitting behind her is reading to her from a book.

Picture 16B: Three hands are grabbing a man dressed in a long coat.

Picture 13B: A boy sitting in the doorway of a log cabin.

Picture 9BM: Four men lying in a field against one another.

Picture 15: A man is standing with his hands clasped together. There are tombstones everywhere.

Picture 12BG: There is a tree and a rowboat next to it in a country setting with no presence of a human being.

Picture 20: The card shows a man leaning against a lamppost at night in a hazy atmosphere.

2 Data collection details

Exclusion criteria for all groups comprised history of a neurological or medical disorder, history of head injury, or alcohol or illicit substance misuse or dependence. Transition to psychosis was defined as the onset of frank psychotic symptoms that did not resolve within a week, corresponding to a severity scale score of 6 on the Disorders of Thought Content subscale, 5 or 6 on the Perceptual Abnormalities subscale and/or 6 on the Disorganized Speech subscales of the CAARMS.

Below we provide more detailed descriptions of the DCT story task and the free speech task.

2.1 DCT story task

For this task, we asked participants to re-tell six stories from the Discourse Comprehension Test (DCT; [2]). The participants were asked to remember what happened in each story. After each story, the participant was asked to re-tell the story to the interviewer, and their response was recorded. Participants were asked to try to mention as many details as possible, including people's names. The task took around 30 minutes in total.

2.2 Free speech task

Participants were asked to speak for 10 minutes on any subject, whilst being audio-recorded. Beforehand, participants were asked what subjects they felt able to talk about. This list of topics was then used by the interviewer to prompt the participant if they stopped talking. Participants' responses covered a wide range of topics, including their plans for the weekend, descriptions of life events and talking about their hobbies and interests.

3 Speech graph measure details

We defined LCC, LSC, LCCr and LSCr as previously proposed by [3, 4]. Namely, the LCC is the largest sub-graph in which all nodes are linked by at least one path. The LSC is the largest sub-graph in which all nodes are linked by a path which can be traversed in either direction (i.e. there is a path from node i to node j, and also from node j to node i). To control for the number of words spoken, LCC and LSC were calculated for windows of 30 words, which overlapped by 15 words, then averaged across all windows [5]. We also calculated values of LCC and LSC normalised to the equivalent measures from randomised speech graphs (obtained by randomly shuffling the

words within each window), to determine how close to randomness the connectedness measures were; denoted LCCr and LSCr [5].

4 Normality

We performed Shapiro-Wilk tests to test the Normality of the NLP measures, for the TAT speech excerpts. Results are shown in Table S1.

NLP measure	Controls	CHR-P	FEP
No. words	0.34	0.61	0.77
No. sent.s	0.61	**0.028**	0.17
Mean sent. length	**<0.001**	0.38	0.24
Coherence	0.74	0.36	0.21
Tangentiality	0.12	0.90	0.21
On-topic	0.91	0.33	0.69
Max. similarity	**0.0088**	0.47	0.22
Ambig. Pronouns	0.21	**0.021**	0.15
LCC	0.40	0.23	0.16
LSC	0.73	0.16	**0.030**
LCCr	0.099	0.81	**0.039**
LSCr	**0.033**	0.78	0.45

Table S1: P-values for Shapiro-Wilk tests to test the Normality of the NLP measures, for the TAT speech excerpts.

5 Antipsychotic medication

To assess whether our results were likely to be driven by group differences in antipsychotic medication, we re-calculated the group differences in NLP measures for the TAT pictures excluding the 4 CHR-P subjects and 6 FEP patients who had been prescribed antipsychotic medication. The results are given in Table S2 and are qualitatively similar to the results in the main text, apart from the FEP/control difference in number of words, which is no longer significant. We therefore conclude that group differences in antipsychotic medication did not explain most of the results observed.

NLP measure	FEP/CON	CHR-P/CON	FEP/CHR-P
No. words	-1.7 (0.081)	-1.5 (0.12)	-0.64 (0.52)
No. sent.s	**2.0 (0.044)**	0.39 (0.70)	1.7 (0.082)
Mean sent. length	**-3.3 (<0.001)**	-1.5 (0.12)	**-2.2 (0.026)**
Coherence	**-3.1 (0.0022)**	**-2.1 (0.034)**	-1.5 (0.14)
Tangentiality	-1.1 (0.28)	-0.72 (0.47)	-0.022 (0.98)
On-topic	**-3.2 (0.0014)**	**-2.7 (0.0061)**	-1.1 (0.26)
Max. similarity	1.4 (0.16)	0.60 (0.55)	1.3 (0.21)
Ambig. Pronouns	0.24 (0.81)	1.5 (0.15)	-1.2 (0.25)
LCC	**-2.9 (0.0033)**	-1.5 (0.13)	**-2.5 (0.011)**
LSC	-1.5 (0.14)	-1.5 (0.14)	-0.81 (0.42)
LCCr	**-3.1 (0.0022)**	-1.8 (0.077)	**-2.3 (0.021)**
LSCr	**-3.1 (0.0017)**	-1.1 (0.27)	**-2.3 (0.021)**

Table S2: Group differences in NLP measures for the TAT pictures after excluding the 4 CHR-P subjects and 6 FEP patients who had been prescribed antipsychotic medication. The Z-values from Mann-Whitney U-tests are given, with the corresponding P-values in brackets.

6 Transition to psychosis

We tested for differences in the NLP measures between the 8 CHR-P subjects who transitioned to psychosis and the 16 CHR-P subjects who did not transition, whilst controlling for IQ, using a GAMLSS model with a Gamma distribution; see Table S3.

NLP measure	T-value	P-value
No. words	-0.20	0.85
No. sent.s	0.19	0.85
Mean sent. length	-0.22	0.83
Coherence	-0.39	0.70
Tangentiality*	0.73	0.48
On-topic	-0.74	0.47
Max. similarity	1.5	0.15
Ambig. Pronouns	-0.43	0.67
LCC	-1.9	0.070
LSC	**-2.8**	**0.011**
LCCr	-1.5	0.16
LSCr	**-3.1**	**0.0050**

Table S3: Group differences in NLP measures for the CHR-P subjects who did and did not transition to psychosis (GAMLSS model, for the TAT picture results), controlling for IQ. *For tangentiality, we used a Gumbel distribution instead of a Gamma distribution, due to negative values.

7 Number of prompts

We calculated the average number of prompts given to each participant when describing the TAT pictures, and tested for group differences using the Mann-Whitney U-test. The results are shown in Table S4.

	Z-value
FEP/CON	2.6 (P=0.0084)
FEP/CHR-P	2.3 (P=0.020)
CHR-P/CON	1.8 (P=0.078)

Table S4: Group differences in the average number of prompts given to each participant when describing the TAT pictures, calculated using the Mann-Whitney U-test.

8 Inaudible pieces of speech

Inaudible pieces of speech were marked as [?] in the transcripts. We counted the number of inaudible pieces of speech per excerpt, then divided by the total number of words in the excerpt to get number of inaudible pieces of speech per word.

The differences in the number of inaudible pieces of speech per word between groups for the TAT, DCT and free speech recordings are shown in Figure S1 and Table S5. For the TAT there was a significant difference in the number of inaudible pieces of speech per word between the CHR-P and control groups, whilst for the DCT there was a significant difference between the FEP and control groups.

	TAT	DCT	Free
FEP/CON	1.1 (P=0.26)	**2.0 (P=0.047)**	0.41 (P=0.68)
FEP/CHR-P	-1.2 (P=0.22)	1.7 (P=0.086)	-0.24 (P=0.81)
CHR-P/CON	**2.2 (P=0.029)**	1.1 (P=0.26)	0.36 (P=0.72)

Table S5: Differences in the number of inaudible pieces of speech per word between the TAT, DCT and free speech recordings, calculated using the Mann-Whitney U-test.

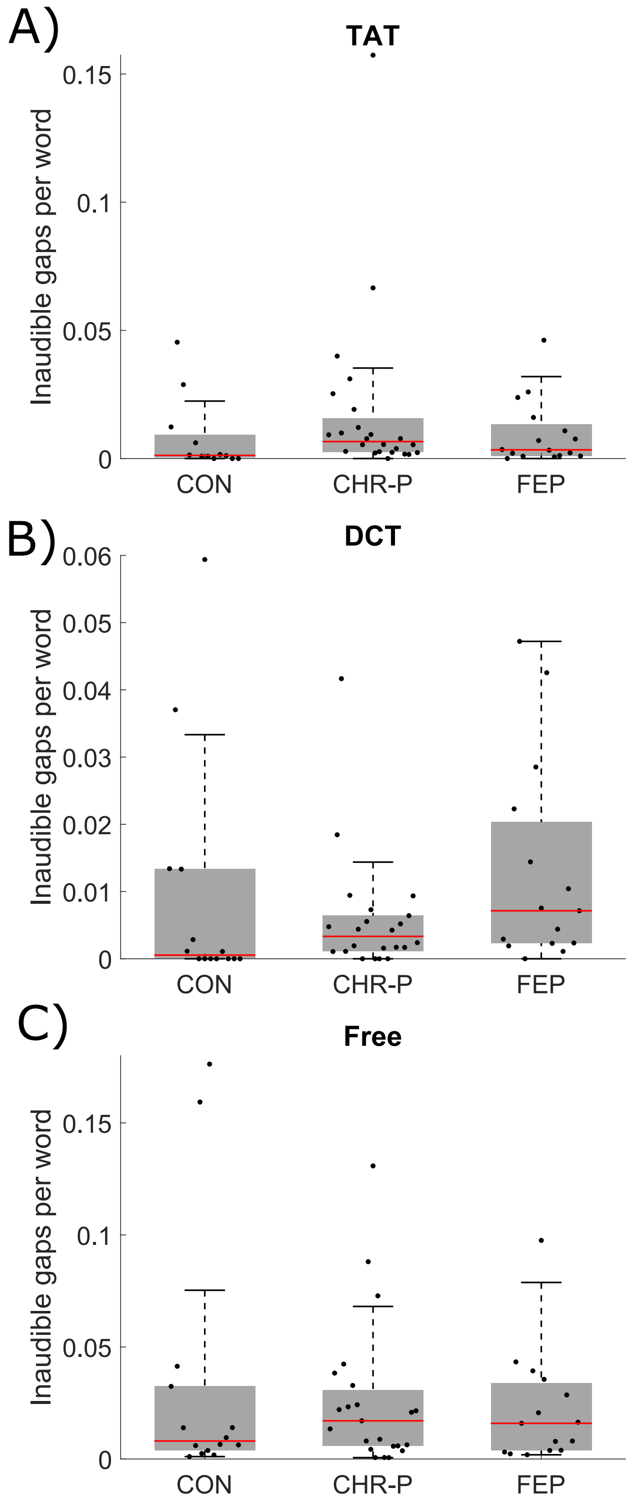

Figure S1: Group differences in number of inaudible pieces of speech per word, for the A) TAT, B) DCT and C) free speech excerpts.

We therefore tested whether the previously identified group differences in the NLP metrics from the TAT remained significant when controlling for the number of inaudible pieces of speech per word. To that end we used a Generalized Additive Model for Location, Scale and Shape (GAMLSS) with a gamma distribution [6], including number of inaudible pieces of speech per word as a co-variate. Results are shown in Table S6. All previously identified significant group differences remained significant.

	FEP/CON	CHR-P/CON	FEP/CHR-P
No. words	-2.1 (P=0.046)	N/A	N/A
No. sentences	2.4 (P=0.024)	N/A	N/A
Sentence length	-3.7 (P=0.0012)	N/A	N/A
Coherence	-4.5 ($P < 0.001$)	-2.3 (P=0.029)	N/A
Tangentiality	N/A	N/A	N/A
On-topic	-5.0 ($P < 0.001$)	-3.4 (P=0.0018)	N/A
Max. similarity	N/A	N/A	N/A
Ambig. pronouns	N/A	N/A	N/A
LCC	-3.9 ($P < 0.001$)	N/A	-3.6 ($P < 0.001$)
LSC	N/A	N/A	N/A
LCCr	-3.9 ($P < 0.001$)	N/A	-3.3 (P=0.0023)
LSCr	-3.4 (P=0.0024)	N/A	-2.8 (P=0.0089)

Table S6: Group differences in NLP metrics from the TAT, assessed using a GAMLSS model with a gamma distribution, controlling for number of inaudible pieces of speech per word as a co-variate.

9 Relationships between NLP measures and the TLI, PANSS scores and cognitive measures

Table S7 shows the associations between the NLP measures and the TLI (TLI total, TLI positive and TLI negative), PANSS symptoms (PANSS positive, PANSS negative and PANSS general), WRAT IQ and number of years in education; for the TAT speech excerpts. We note that all subjects had data available for the TLI, IQ and number of years in education, whilst 15 CHR-P subjects, 8 FEP patients and no control subjects had PANSS data available.

NLP Measure	TLI total	TLI positive	TLI negative	PANSS positive	PANSS negative	PANSS general	WRAT IQ	Years education
No. words	0.11 (0.97)	1.6 (0.46)	**-4.9 (< 0.001)**	1.0 (0.65)	-0.38 (0.89)	0.18 (0.96)	-0.28 (0.94)	-1.2 (0.54)
No. sent.s	0.22 (0.95)	0.42 (0.89)	-0.54 (0.85)	1.2 (0.54)	-0.26 (0.94)	0.54 (0.85)	-2.9 (0.077)	-1.4 (0.47)
Mean sent.	0.0028 (1.0)	0.89 (0.73)	-2.5 (0.13)	-0.14 (0.97)	-0.58 (0.85)	-0.54 (0.85)	1.5 (0.46)	-0.12 (0.97)
Coherence	-0.48 (0.87)	0.29 (0.94)	-1.9 (0.39)	-0.37 (0.89)	-1.0 (0.65)	-0.60 (0.85)	1.2 (0.52)	0.82 (0.77)
Tangentiality	-0.44 (0.88)	0.26 (0.94)	-1.9 (0.39)	-0.53 (0.85)	0.0064 (1.0)	-1.2 (0.54)	-0.51 (0.86)	2.5 (0.13)
On-topic	-2.8 (0.077)	-2.0 (0.33)	-1.8 (0.41)	-1.5 (0.46)	-1.8 (0.43)	-0.78 (0.78)	1.7 (0.46)	1.5 (0.46)
Max. similarity	1.0 (0.65)	0.98 (0.65)	-0.027 (1.0)	-1.3 (0.52)	-1.4 (0.49)	-1.3 (0.50)	-1.4 (0.46)	-1.4 (0.47)
Ambig. pronouns	1.1 (0.59)	1.6 (0.46)	-1.5 (0.46)	1.5 (0.46)	-0.43 (0.89)	1.6 (0.46)	-0.12 (0.97)	0.0083 (1.0)
LCC	-0.72 (0.80)	0.70 (0.81)	**-4.1 (0.0038)**	0.76 (0.78)	-0.56 (0.85)	0.083 (0.99)	1.7 (0.43)	1.9 (0.39)
LSC	-2.1 (0.27)	-0.39 (0.89)	**-5.4 (0.00018)**	-0.21 (0.95)	-1.2 (0.54)	-0.41 (0.89)	2.6 (0.13)	1.6 (0.46)
LCCr	-0.76 (0.78)	0.76 (0.78)	**-4.4 (0.0023)**	0.21 (0.95)	-0.62 (0.85)	-0.56 (0.85)	1.5 (0.46)	1.6 (0.46)
LSCr	-1.3 (0.51)	0.042 (1.0)	**-3.6 (0.014)**	0.59 (0.85)	-0.86 (0.76)	0.26 (0.94)	2.9 (0.077)	2.4 (0.15)

Table S7: Supplementary Table S1 shows the associations between the NLP measures and the TLI (TLI total, TLI positive and TLI negative), PANSS symptoms (PANSS positive, PANSS negative and PANSS general), WRAT IQ, and number of years in education. Results are shown as T-statistics, with FDR corrected P-values in brackets (corrected for $12 \times 8 = 96$ multiple comparisons.

10 Education and IQ

We tested whether the previously identified group differences in the NLP metrics from the TAT remained significant when controlling for number of years in education and IQ. To that end we used a Generalized Additive Model for Location, Scale and Shape (GAMLSS) with a gamma distribution [6], including number of years in education or IQ as co-variates. Results are shown in Tables S8 and S9 respectively. As well as T-statistics and P-values, we also report multiplicative effect sizes on the mean, λ. Here, λ is given as the exponent of the μ coefficient of the GAMLSS model. For example, λ=0.8 would correspond to a 20% decrease in the mean value of the NLP metric in the FEP patients compared to the control subjects.

	FEP/CON	CHR-P/CON	FEP/CHR-P
No. words	T=-1.4, P=0.18, λ=0.91	N/A	N/A
No. sentences	T=1.5, P=0.15, λ=1.1	N/A	N/A
Sentence len.	**T=-2.7, P=0.012, λ=0.77**	N/A	N/A
Coherence	**T=-2.5, P=0.021, λ=0.94**	T=-1.6, P=0.12, λ=0.91	N/A
Tangentiality	N/A	N/A	N/A
On-topic	**T=-2.8, P=0.0093, λ=0.95**	T=-1.9, P=0.065, λ=0.94	N/A
Max. sim.	N/A	N/A	N/A
Ambig. pron.	N/A	N/A	N/A
LCC	**T=-2.2, P=0.035, λ=0.97**	N/A	**T=-4.0, P$<$ 0.001, λ=0.95**
LSC	N/A	N/A	N/A
LCCr	**T=-2.1, P=0.044, λ=0.99**	N/A	**T=-3.4, P=0.0015, λ=0.98**
LSCr	T=-1.8, P=0.091, λ=0.95	N/A	**T=-3.5, P=0.0013, λ=0.88**

Table S8: Group differences in NLP metrics from the TAT, assessed using a GAMLSS model with a gamma distribution, controlling for years in education as a co-variate. Results are given as T-statistics, P-values and multiplicative effect sizes (λ).

	FEP/CON	CHR-P/CON	FEP/CHR-P
No. words	T=-1.7, P=0.11, λ=0.90	N/A	N/A
No. sentences	T=1.1, P=0.28, λ=1.1	N/A	N/A
Sentence len.	**T=-2.2, P=0.038, λ=0.82**	N/A	N/A
Coherence	**T=-3.2, P=0.0039, λ=0.92**	T=-1.4, P=0.18, λ=0.93	N/A
Tangentiality	N/A	N/A	N/A
On-topic	**T=-3.0, P=0.0065, λ=0.95**	**T=-2.3, P=0.026, λ=0.93**	N/A
Max. sim.	N/A	N/A	N/A
Ambig. pron.	N/A	N/A	N/A
LCC	**T=-2.2, P=0.035, λ=0.97**	N/A	**T=-3.5, P=0.0014, λ=0.95**
LSC	N/A	N/A	N/A
LCCr	**T=-2.4, P=0.024, λ=0.99**	N/A	**T=-2.8, P=0.0089, λ=0.98**
LSCr	T=-1.7, P=0.096, λ=0.95	N/A	**T=-2.8, P=0.0088, λ=0.90**

Table S9: Group differences in NLP metrics from the TAT, assessed using a GAMLSS model with a gamma distribution, controlling for IQ as a co-variate. Results are given as T-statistics, P-values and multiplicative effect sizes (λ).

11 Digit span test scores

Table S10 shows the relationships between the digit span test scores and the 12 NLP metrics, for the TAT task. We observed a significant correlation between the digit span test score and semantic coherence.

We tested whether the previously identified group differences in the NLP metrics remained significant when controlling for the digit span test score. To that end we used a Generalized Additive Model for Location, Scale and Shape (GAMLSS) with a gamma distribution [6], including the digit span test score as a co-variate. Results are shown in Table S11. Group differences in on-topic score and speech graph connectivity measures remained significant.

NLP metric	T-stat	P-value (FDR)
No. words	1.4	0.25
No. sentences	-1.2	0.30
Sentence length	1.5	0.23
Coherence	**3.1**	**0.041**
Tangentiality	0.11	0.91
On-topic	2.3	0.16
Max. similarity	-1.2	0.30
Ambig. pronouns	-0.53	0.65
LCC	1.7	0.21
LSC	1.7	0.21
LCCr	2.0	0.20
LSCr	1.7	0.21

Table S10: Associations between the NLP measures and digit span test scores, for the TAT task. Results are shown as T-statistics, with FDR corrected P-values in brackets (corrected for 12 multiple comparisons).

	FEP/CON	CHR-P/CON	FEP/CHR-P
No. words	T=-0.34, P=0.74, λ=0.98	N/A	N/A
No. sentences	T=1.3, P=0.21, λ=1.1	N/A	N/A
Sentence len.	T=-1.5, P=0.15, λ=0.87	N/A	N/A
Coherence	T=-1.9, P=0.078, λ=0.95	T=-1.1, P=0.29, λ=0.96	N/A
Tangentiality	N/A	N/A	N/A
On-topic	**T=-2.2, P=0.043, λ=0.96**	**T=-2.4, P=0.023, λ=0.93**	N/A
Max. sim.	N/A	N/A	N/A
Ambig. pron.	N/A	N/A	N/A
LCC	**T=-2.8, P=0.010, λ=0.97**	N/A	**T=-3.4, P=0.0018, λ=0.95**
LSC	N/A	N/A	N/A
LCCr	**T=-2.8, P=0.011, λ=0.99**	N/A	**T=-2.6, P=0.014, λ=0.98**
LSCr	**T=-2.3, P=0.035, λ=0.94**	N/A	**T=-2.2, P=0.029, λ=0.92**

Table S11: Group differences in NLP metrics from the TAT, assessed using a GAMLSS model with a gamma distribution, controlling for digit span test score as a co-variate. Results are given as T-statistics, P-values and multiplicative effect sizes (λ).

12 DCT results

12.1 Digit span test scores

Table S12 shows the relationships between the digit span test scores and the 12 NLP metrics, for the DCT task. We observed significant correlations between the digit span test score and number of sentences, on-topic score and ambiguous pronoun count.

NLP metric	T-stat	P-value (FDR)
No. words	-2.0	0.095
No. sentences	**-2.8**	**0.0496**
Sentence length	1.3	0.31
Coherence	2.0	0.095
Tangentiality	0.0070	0.99
On-topic	**2.6**	**0.0496**
Max. similarity	-0.77	0.54
Ambig. pronouns	**-3.0**	**0.0496**
LCC	1.1	0.40
LSC	2.1	0.095
LCCr	0.67	0.55
LSCr	2.0	0.095

Table S12: Associations between the NLP measures and digit span test scores, for the DCT task. Results are shown as T-statistics, with FDR corrected P-values in brackets (corrected for 12 multiple comparisons).

We tested whether the previously identified group differences in the NLP metrics from the DCT remained significant when controlling for the digit span test score. To that end we used a Generalized Additive Model for Location, Scale and Shape (GAMLSS) with a gamma distribution [6], including the digit span test score as a co-variate. Results are shown in Table S13. No NLP group differences remained significant.

	FEP/CON	CHR-P/CON	FEP/CHR-P
No. words	T=-1.5, P=0.15, λ=0.84	N/A	N/A
No. sentences	N/A	N/A	N/A
Sentence len.	T=-0.80, P=0.43, λ=0.91	N/A	T=-1.9, P=0.071, λ=0.74
Coherence	T=-1.7, P=0.11, λ=0.96	N/A	T=-1.7, P=0.10, λ=0.93
Tangentiality	N/A	N/A	N/A
On-topic	T=-1.5, P=0.14, λ=0.97	N/A	T=-1.8, P=0.075, λ=0.94
Max. sim.	N/A	N/A	N/A
Ambig. pron.	*	N/A	N/A
LCC	T=-1.8, P=0.083, λ=0.98	N/A	T=-1.6, P=0.13, λ=0.97
LSC	T=-1.7, P=0.25, λ=0.98	N/A	T=-1.6, P=0.13, λ=0.95
LCCr	T=-1.7, P=0.11, λ=0.99	N/A	N/A
LSCr	T=-1.9, P=0.067, λ=0.94	T=-1.5, P=0.15, λ=0.96	T=-2.0, P=0.053, λ=0.91

Table S13: Group differences in NLP metrics from the DCT, assessed using a GAMLSS model with a gamma distribution, controlling for digit span test score as a co-variate. Results are given as T-statistics, P-values and multiplicative effect sizes (λ). *We note that we were unable to test for a group difference in ambiguous pronoun count between the FEP patients and control subjects- see Section 8 for details.

12.2 Inaudible pieces of speech

We tested whether the previously identified group differences in the NLP metrics from the DCT remained significant when controlling for the number of inaudible pieces of speech per word. To that end we used a Generalized Additive Model for Location, Scale and Shape (GAMLSS) with a gamma distribution [6], including number of inaudible pieces of speech per word as a co-variate. Results are shown in Table S14. All previously identified significant group differences remained

significant, apart from the FEP/CON group difference in number of words. We also note that we were unable to test for a group difference in ambiguous pronoun count between the FEP patients and control subjects using the GAMLSS model due to some subjects having ambiguous pronoun counts of 0 (which is incompatible with using a gamma distribution).

	FEP/CON	CHR-P/CON	FEP/CHR-P
No. words	-0.28 (P=0.78)	N/A	N/A
No. sentences	N/A	N/A	N/A
Sentence length	-2.4 (P=0.025)	N/A	-2.2 (P=0.035)
Coherence	-3.6 (P=0.0015)	N/A	-2.3 (P=0.028)
Tangentiality	N/A	N/A	N/A
On-topic	-4.0 (P< 0.001)	N/A	-2.2 (P=0.034)
Max. similarity	N/A	N/A	N/A
Ambig. pronouns	*	N/A	N/A
LCC	-3.5 (P=0.0020)	N/A	-2.5 (P=0.019)
LSC	-3.3 (P=0.0026)	N/A	-2.1 (P=0.043)
LCCr	-3.0 (P=0.0062)	N/A	N/A
LSCr	-4.3 (P< 0.001)	-2.6 (P=0.026)	-3.0 (P=0.0055)

Table S14: Group differences in NLP metrics from the DCT, assessed using a GAMLSS model with a gamma distribution, controlling for number of inaudible pieces of speech per word as a co-variate. *We note that we were unable to test for a group difference in ambiguous pronoun count between the FEP patients and control subjects- see text for details.

The differences in the number of inaudible pieces of speech per word between the TAT, DCT and free speech recordings are shown in Figure S2 and Table S15.

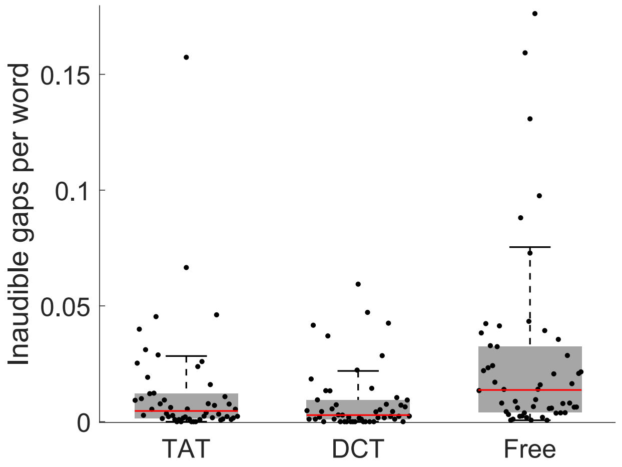

Figure S2: Number of inaudible pieces of speech per word, for the TAT, DCT and free speech excerpts.

	Z-value	P-value
TAT/DCT	1.2	0.23
TAT/Free	-3.1	0.0022
DCT/Free	-4.0	< 0.001

Table S15: Differences in the number of inaudible pieces of speech per word between the TAT, DCT and free speech recordings, calculated using the Mann-Whitney U-test.

References

[1] P. Shrestha, "Detailed Procedure of Thematic Apperception test," 2017.

[2] R. Brookshire and L. Nicholas, "The discourse comprehension test." Tucson, AZ: Communication Skill Builders/The Psychological Corporation, 1993.

[3] N. Mota, N. Vasconcelos, N. Lemos, A. Pieretti, O. Kinouchi, G. Cecchi, M. Copelli, and S. Ribeiro, "Speech graphs provide a quantitative measure of thought disorder in psychosis," *PLoS ONE*, vol. 7, p. e34928, 2012.

[4] N. B. Mota, R. Furtado, P. Maia, M. P. C., Copelli, and S. Ribeiro, "Graph analysis of dream reports is especially informative about psychosis," *Scientific Reports*, vol. 4, p. 3691, 2014.

[5] T. Spencer, B. Thompson, D. Oliver, K. Dierderen, A. Demjaha, S. Morgan, S. Weinstein, D. Stahl, F. Day, L. Valmaggia, G. Rutigliano, A. D. Micheli, N. B. Mota, P. Fusar-Poli, and P. McGuire, "Lower speech connectedness linked to incidence of psychosis in people at clinical high risk," *Schizophrenia Research*, 2020.

[6] R. Rigby, D. Stasinopoulos, and P. Lane, "Generalized additive models for location, scale and shape," *J. R. Stat. Soc. Ser. C Appl. Stat.*, vol. 54, pp. 507–554, 2005.
